# De Garengeot’s hernia: A rare presentation of acute appendicitis and challenging preoperative diagnosis

**DOI:** 10.1016/j.radcr.2026.06.058

**Published:** 2026-07-04

**Authors:** Peter Vo, Rishma Gattu, Rishabh Gattu

**Affiliations:** a Department of Medical Imaging, Arrowhead Regional Medical Center, Colton, CA; b School of Medicine, Mercy Fitzgerald Hospital, Darby, PA

**Keywords:** De Garengeot’s hernia, Femoral hernia, Acute appendicitis

## Abstract

De Garengeot hernias are femoral hernias that contain the appendix. They represent a rare subtype of femoral hernias, which are themselves a small portion of all inguinal hernias. A 55-year-old female with a history of type 2 diabetes mellitus complaining of right inguinal bulge and acute onset right lower quadrant pain. Computed tomography (CT) showed right groin soft tissue mass and surrounding fat inflammation within the femoral sac, consistent with incarcerated appendix. The patient underwent urgent appendectomy with hernia repair and was discharged from the hospital without further complication. De Garengeot hernias have nonspecific presentations but may develop severe complications such as incarceration, strangulation, and bowel necrosis. Preoperative diagnosis is paramount in prompt treatment.

## Introduction

First described by René Jacques Croissant de Garengeot in 1731, de Garengeot hernias are characterized by the presence of an inflamed appendix within a femoral hernia sac. A hernia is defined as the protrusion of tissue through a defect in the surrounding connective structures, typically occurring in the groin. Of all groin hernias, approximately 3% are femoral [1]. Within this subset, 1% contain the appendix, with only up to 0.13% containing acute appendicitis [2]. We present a case of de Garengeot hernia diagnosed preoperatively via CT. We chose this case to highlight the importance of preoperative diagnosis in differentiating de Garengeot hernias from other sources of abdominal pain.

## Case report

A 55-year-old female with a history of type 2 diabetes mellitus presented to the emergency department complaining of a right inguinal bulge, acute onset right lower quadrant pain, and nausea for 1 day after mild physical activity. She denied any history of hernia or surgery in the past.

On admission, the patient was noted to be in acute distress, but was afebrile with a normal heart rate and rhythm. Abdominal exam revealed right upper and lower quadrant tenderness with guarding. No rebound or peritonism was elicited. A small mass was felt in the right inguinal area without overlying skin changes. Complete blood count, comprehensive metabolic panel, and urinalysis were unremarkable. Differential diagnosis at this time included intra-abdominal perforation, acute appendicitis, and colitis.

CT scan of the abdomen and pelvis with intravenous contrast was ordered. Examination revealed a right groin soft tissue mass with surrounding fat inflammation (Fig. 1). Further evaluation demonstrated that this mass originated from the cecum (Fig. 2) and was located medially to the femoral vein (Fig. 3), confirming that this was an inflamed appendix herniating into the femoral canal. The appendix was dilated to 8 mm and contained intraluminal fluid. The hernia sac also indented the right femoral vein near the inguinal ligament. Subcentimeter reactive right inguinal lymph nodes were seen. Ultrasound was not performed for this patient.

**Fig. 1:**
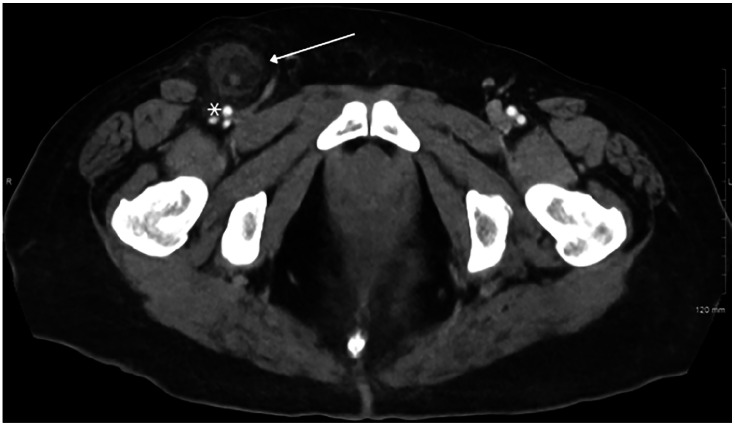
Axial CT image shows an enhancing soft tissue mass (arrow) containing fluid within the right groin, as well as inflammation of the surrounding fat. This mass lies anterior of the femoral vessels (star).

**Fig. 2:**
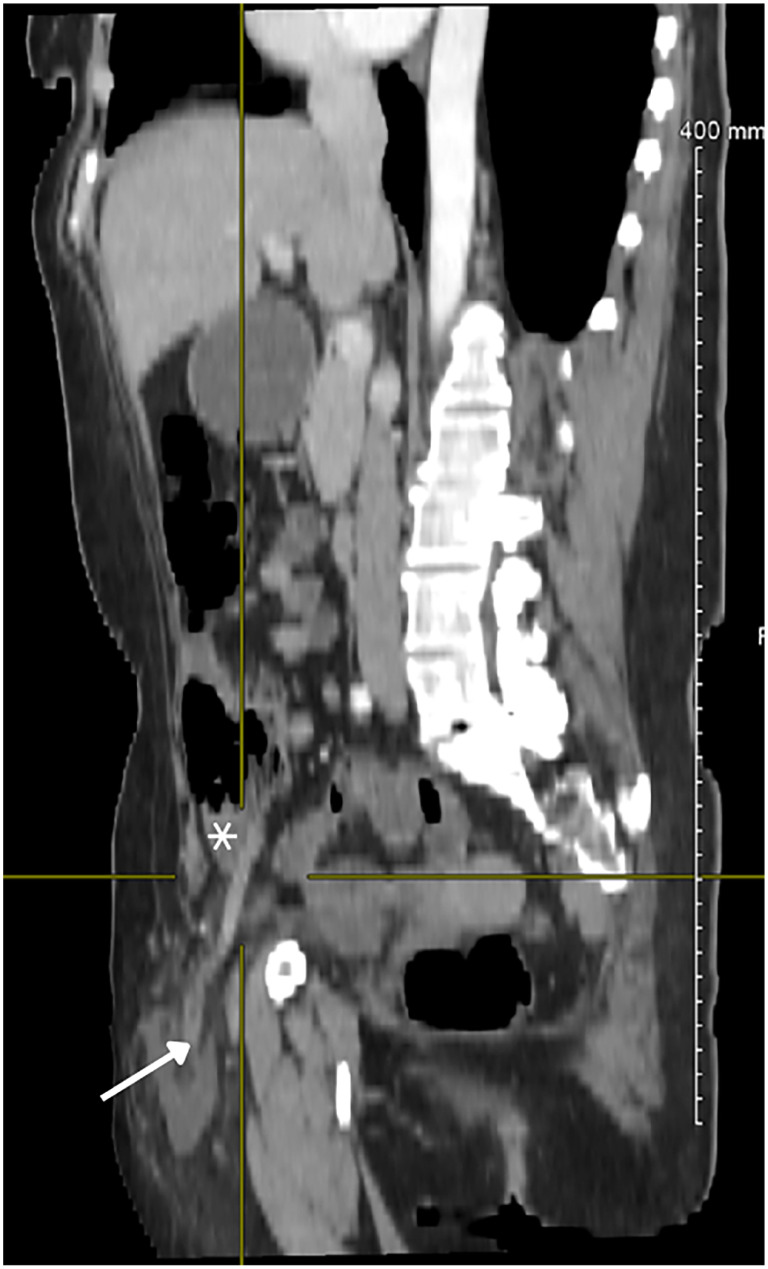
Sagittal multiplanar reformatted shows the soft tissue mass originates from the cecum (star) and ends as a blind tubular structure (arrow), consistent with a herniated appendix.

**Fig. 3:**
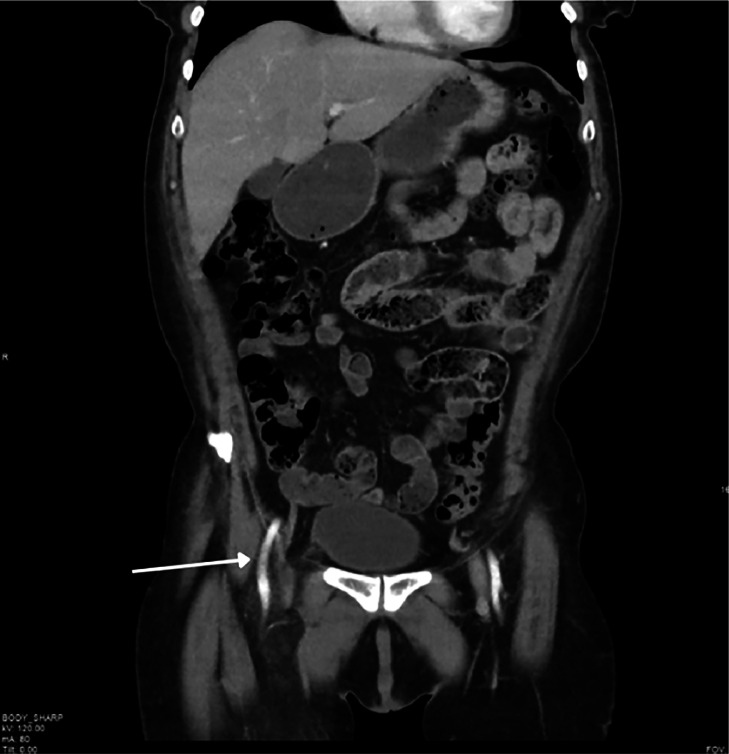
Coronal multiplanar reformat shows the herniated appendix is medial to the right femoral vein (arrow).

General surgery was consulted, and the patient underwent laparoscopic appendectomy and open right femoral hernia repair. Final pathology confirmed acute suppurative appendicitis with periappendicitis and an incidentally associated round ligament lipoma.

## Discussion

Recent literature suggests that only 31.5% of all de Garengeot hernias were diagnosed pre-operatively, with a sensitivity of 61% on preoperative CT scan. Notably, 81% of cases were observed in females, with increasing rates of diagnosis in the past decade [3]. These hernias should remain distinct from Amyland hernias, which are a rare form of inguinal hernia in which the appendix is located within the herniated sac.

Preoperative diagnosis of de Garengeot hernias can be challenging as symptoms can be nonspecific, such as right lower quadrant pain with groin mass [4]. To further obscure accuracy, differential diagnoses can be broad and can include, but are not limited to, fat necrosis, lymphadenopathy, or abscesses [5]. Fat necrosis would demonstrate ovoid fat attenuation, lymphadenopathy would show lobulations and other amorphous characteristics, and abscesses would display complicated fluid collection. However, the key finding for distinguishing de Garengeot hernias from other pathologies would be delineating that the soft tissue mass originates from the cecum. Preoperative CT can aid in differentiating these diagnoses and assessing for other acute complications.

Femoral hernias demonstrate a higher rate of incarceration (up to 56 %) compared to inguinal hernias (up to 10 %). The most serious complication is strangulation, which occurs at a rate of up to 20% [6]. While typical appendicitis involves appendiceal dilatation and inflammation of the wall as a result of intrinsic obstruction (ie, appendicolith and hypertrophied lymphatic tissue), de Garengeot hernias are often secondary to mechanical obstruction at the neck of the hernia [7]. Definitive treatment is prompt surgical intervention, though the best approach has not yet been standardized [8]. In general, laparoscopic methods are favored [9].

## Conclusion

De Garengeot hernia remains an elusive diagnosis. In most cases, the diagnosis is only accurately assessed via surgical intervention. While a rare etiology of abdominal pain, such hernias should remain in the differential diagnosis of RLQ pain, especially in female patients presenting with an inguinal mass. Preoperative CT imaging allows for timely and accurate diagnosis, in an effort to avoid severe complications such as strangulation.

## Patient consent

The patient has provided informed consent for the publication of this case report.
